# Protective effect of dexmedetomidine in coronary artery bypass grafting surgery

**DOI:** 10.3892/etm.2013.1183

**Published:** 2013-06-25

**Authors:** JIANJUN REN, HUIJUN ZHANG, LINING HUANG, YUE LIU, FENGQIN LIU, ZHENMING DONG

**Affiliations:** 1 Department of Anesthesiology, The Second Affiliated Hospital, Hebei Medical University, Shijiazhuang, Hebei 050000;; 2 Dezhou Institute for Drug Control, Dezhou, Shandong 253015, P.R. China

**Keywords:** dexmedetomidine, hemodynamics, cardiac function, myocardial reperfusion injury, coronary artery bypass grafting, off-pump

## Abstract

The aim of this study was to observe the impact of dexmedetomidine on postoperative myocardial injury in patients undergoing off-pump coronary artery bypass (OPCAB) grafting. One hundred and sixty-two patients who were undergoing OPCAB surgery were randomly divided into control and dexmedetomidine groups (groups C and Dex, respectively). Following the first vascular anastomosis grafting, the patients in group Dex received a continuous intravenous infusion of 0.2–0.5 *μ*g/kg/h dexmedetomidine, until they were transferred to the Cardiac Surgery intensive care unit (ICU) for 12 h. Patients in group C received physiological saline intraoperatively and an intravenous infusion of 2–4 mg/kg/h isopropylphenol for postoperative sedation. Invasive arterial pressure and heart rate were continuously monitored for 5 min subsequent to entry into the operating theatre (T0), immediately following surgery (T1), 12 h post-surgery (T2), 24 h post-surgery(T3), 48 h post-surgery(T4) and 72 h post-surgery (T5). Blood samples were taken to determine the plasma levels of cardiac troponin I (cTnI) and creatine kinase-MB (CK-MB) at each time point. At 72 h post-surgery, a dynamic electrocardiogram was monitored. The blood pressure, heart rate, levels of cTnI, CK-MB, norepinephrine and cortisol, and postoperative arrhythmic events in the patients in group Dex all decreased compared with those in group C. The duration of mechanical ventilation and ICU residence time were also shorter than those in the control group (P<0.05). Dexmedetomidine reduced post-surgical myocardial injury in patients who had undergone OPCAB surgery.

## Introduction

Coronary artery bypass grafting (CABG) is an effective method of treating coronary artery stenosis. The traditional surgical approach is performed under cardiac arrest with cardiopulmonary bypass (CPB), which has the potential to result in myocardial injuries, such as increased levels of cardiac troponin I (cTnI) and creatine kinase-MB (CK-MB) (1). CTnI and CK-MB levels may therefore be indicative of the result of the surgery. The three-year survival rates of patients whose cTnI and CK-MB levels were above the threshold value have been demonstrated to be lower than those of patients with normal cTnI and CK-MB levels (2). With the development of fixation technology, the traditional surgical approach has gradually been replaced by off-pump coronary artery bypass (OPCAB) grafting (3). OPCAB surgery may reduce, although not entirely eliminate, post-surgical myocardial injuries (4,5). Eliminating or reducing these myocardial injuries is likely to improve the prognosis in patients who have undergone cardiac surgery (4). Therefore, various measures have been clinically applied to reduce the incidence of post-surgical myocardial injuries (6–8).

For many years, α_2_-adrenergic receptor agonists that confer myocardial protection have been administered to patients with heart diseases in non-cardiac and cardiac surgeries (9,10). Dexmedetomidine is a novel type of α_2_-adrenergic receptor agonist with a highly selective application, and is predominantly used as a sedative or adjuvant anesthetic drug in clinical settings (11–13). From clinical observation, dexmedetomidine has been revealed to reduce the incidence of cardiovascular adverse events in patients with heart diseases during non-cardiac surgeries (14). As early as 1997, the impact of dexmedetomidine on post-surgical myocardial ischemia in patients who had received on-pump coronary artery bypass grafting was observed, although the results of the observation did not reveal any reduction in myocardial ischemic events (15). Although dexmedetomidine has been administered to patients undergoing cardiac surgery, its use has been limited to postoperative sedation (16,17) and the treatment of arrhythmia in cardiac surgery (18–20). No results are available concerning the impact of the intraoperative application of dexmedetomidine on post-surgical myocardial injury in patients who have undergone OPCAB surgery. We hypothesized that dexmedetomidine had the potential to reduce postoperative myocardial injuries in patients who had undergone OPCAB surgery. Myocardial injuries were assessed by observing the hemodynamic changes, myocardial enzyme levels, myocardial ischemia and arrhythmic events in patients. The data were used as a basis for the application of dexmedetomidine in OPCAB surgery.

## Materials and methods

### Case selection

One hundred and sixty-two patients who were undergoing OPCAB surgery between January 2010 and January 2011 were randomly divided into control and dexmedetomidine-treated groups (groups C and Dex, respectively). The study was conducted in accordance with the Declaration of Helsinki, and with approval from the Ethics Committee of The Second Affiliated Hospital, Hebei Medical University (Shijiazhuang, China). All patients were fully informed about the trial and provided signed consent preoperatively.

### Inclusion criteria

Patients were selected for the study if they were undergoing optional OPCAB surgery, and exhibited three or four coronary artery lesions and sinus rhythm without heart failure. In addition, inclusion criteria comprised normal preoperative liver/kidney kinetic energies, and indicators demonstrating that levels of cTnI and myocardial enzymes were within the normal ranges.

### Exclusion criteria

Patients who were >75 years of age, demonstrated an ejection fraction of <40% or bradycardia based on preoperative diagnosis (HR <50 times/min), and who had a preoperative history of arrhythmia were excluded from the study. In addition, exclusion criteria comprised a preoperative systolic pressure <90 mmHg, obesity, type I or type II diabetes mellitus, drug dependence and a history of psychiatry and cerebrovascular diseases. Any additional medication, with the exception of salicylic acid drugs, was able to be taken until the morning prior to the surgery.

### Surgical methods and medication

Anesthesia was induced in all patients by the administration of 1–2 mg/kg propofol (cat. no.: 1005123; Xi’an Libang Pharmaceutical Co., Ltd., Xi’an, China), 20 *μ*g/kg fentanyl (cat. no.: 2111001; Yichang Humanwell Pharmaceutical Co., Ltd., Yichang, China) and an intravenous injection of 0.1 mg/kg vecuronium bromide (cat. no.: 1007012; Zhejiang Xianju Pharmaceutical Co., Ltd., Taizhou, China). After 3–4 min, patients underwent oral endotracheal intubation. Intraoperatively, patients were administered 2–6 mg/kg/h propofol for sedation, and up to 30 *μ*g/kg fentanyl prior to the sawing open of the sternum. All patients underwent cardiothoracic surgery through a median sternotomy incision. Following the sawing of the sternum, the left internal thoracic arteries were separated, whilst, simultaneously, the great saphenous veins of patients in another group were separated for standby application. Following the separation of the internal left thoracic arteries, the patients were treated with 3 mg/kg heparin sodium. The activated clotting time (ACT) was measured 5 min later, and vascular anastomosis was performed a further 300 sec later. Protamine was used for post-surgical neutralization.

Following the first vascular anastomosis grafting, the patients in group Dex received a continuous intravenous infusion of 0.2–0.5 *μ*g/kg/h dexmedetomidine (cat. no.: 11122034; Jiangsu Hengrui Medicine Co., Ltd., Lianyungang, China) until they were transferred to the Cardiac Surgery intensive care unit (ICU) for 12 h. During the drug administration, the dosages of anesthetics and dexmedetomidine were adjusted according to the hemodynamic changes in the patient, and vasoactive drugs were used when necessary. The patients in group C were administered an intravenous infusion of 0.9% physiological saline, using identical infusion times and methods to group Dex. Following transferral to the Cardiac Surgery ICU, the patients received an intravenous infusion of 2–4 mg/kg/h propofol for sedation. The infusion dosage was adjusted according to hemodynamic changes. Vasoactive drugs and diuretics were used in the ICU based on clinical drug indications.

### Detection indices

Following entry into the operating theater, the patients underwent radial artery cannulation under local anesthesia, and the invasive arterial pressure was monitored. Mean arterial pressure and heart rate were recorded for 5 min following the cannulation, in order to provide reference values (T0). In addition, the mean arterial pressure and heart rate were monitored immediately following surgery (T1), 12 h post-surgery (T2), 24 h post-surgery (T3), 48 h post-surgery (T4) and 72 h post-surgery (T5). At each time point, ∼2 ml arterial blood was collected and centrifuged at 1,500 × g. The serum was then separated and stored at −70°C, prior to the measurement of the serum levels of cTnI and CK-MB, via enzyme-linked immunosorbent assay (ELISA). Plasma norepinephrine and cortisol levels were measured by HPLC.

At 72 h post-surgery, a dynamic electrocardiogram was monitored and recorded for the analysis of postoperative myocardial ischemia and arrhythmia.

### Statistical analysis

SAS statistical software, version 8.15 (SAS Institute, Inc., Cary, NC, USA), was used for the statistical analysis. The normally distributed data are presented as the mean ± standard deviation, and were subjected to repeated measurement design. The data were analyzed using repeated measures analysis of variance (ANOVA), and the enumeration data were analyzed using ANOVA. P<0.05 was considered to indicate a statistically significant difference.

## Results

### Comparison of the preoperative basic characteristics of the patients in the two groups

No differences were observed between the two groups with regard to the preoperative basic characteristics of the patients, including age, gender distribution and the incidence of hypertension and hypercholesterolemia. In addition, there were no significant differences between the patients in the two groups with regard to any preoperative medication taken by the patients, such as β-receptor inhibitors, nitrates and calcium ion antagonists (Table I).

### Comparison of surgical characteristics

There were no patient mortalities in the two groups. The durations of surgery were 2.58 ± 0.23 and 3.12 ± 0.30 h in groups C and Dex, respectively, while the numbers of vascular grafts were 3.38 ± 0.53 and 3.28 ± 0.40 in groups C and Dex, respectively. There were no significant differences between the groups for either of these results (P>0.05).

### Changes in blood pressure and heart rate

At T1 and T2, the blood pressure and heart rate in group Dex were significantly lower than those in group C (P<0.05). No statistical differences were observed between the two groups at the other time points (P>0.05). Systolic blood pressure (SBP) and diastolic blood pressure (DBP) at T1 and T2 were significantly lower than it at T0 in groups C and Dex (P<0.05). Compared with group C, SBP and DBP at T1 and T2 were significantly lower in group Dex (P<0.05). Heart rate (HR) at T1 to T5 were higher than that at T0 in group C. HR at T1 and T2 were significantly lower than that at T0 in group Dex and those at T1 and T2 in group C (P<0.05; Table II).

### Plasma levels of norepinephrine and cortisol

The norepinephrine and cortisol levels at T1 and T2 in group Dex were significantly lower than those in group C (P<0.05). However, there were no statistical differences between the groups at the other time points (P>0.05). In group C, the norepinephrine levels at T1 to T5 were significantly higher than that at T0 (P<0.05). The norepinephrine levels at T1 and T2 were significantly lower than those at T0 and T3–5 in group Dex (P<0.05), which were also significantly lower than those at T1 and T2 in group C (P<0.05). The cortisol levels at T1 and T2 were significantly lower than those at the other time points in groups C and Dex. In addition, the cortisol levels at T1 and T2 in group Dex were significantly lower than those in group C (P<0.05; Table III).

### Concentrations of cTnI and CK-MB

The basic levels of cTnI and CK-MB in the two groups were within the normal ranges, i.e. <0.2 and <4.5 ng/m, respectively. The cTnI and CK-MB levels at T1-5 were higher than those at T0 in the two groups. At T1 and T2, the cTnI and CK-MB levels in group Dex were significantly lower than those in group C (P<0.05). No statistically significant differences were observed at the other time points (P>0.05, Table IV).

### Statistics of arrhythmic events

With regard to atrial fibrillation, there was a significantly greater number of cases in group C than in group Dex (five cases and one case, respectively; P<0.05). In addition, there was a statistically significant difference in the number of cases of supraventricular tachycardia between groups C and Dex (12 and two cases, respectively; P<0.05). Furthermore, there was a significant difference in ventricular tachycardia between groups C and Dex, with six cases and one case, respectively (P<0.05). However, there were no statistically significant differences in premature ventricular contraction between groups C and Dex (nine and 10 cases, respectively; P>0.05).

### Statistics of myocardial ischemic events

The definition of an ST segment event included an ST-segment elevation of 2.0 mm or an ST-segment depression of 1.0 mm (duration, ≥60 sec; interval time, ≥180 sec). There was a statistically significant difference between the number of cases in groups C and Dex (15 and six cases, respectively; P<0.05).

## Discussion

CTnI and CK-MB are recognized as indicators that are predictive of myocardial ischemia injuries. Within 20 h subsequent to surgery, the increase in cTnI level is an independent factor that is predictive of the post-surgical hospital mortality of a patient. Furthermore, the cTnI level may be used to predict the prognosis of patients and the occurrence of two-year post-surgical cardiac ischemic events (21–23). CK-MB is not a specific myocardial injury indicator, but it exhibits an identical function to cTnI when its concentration is >20 IU (2,24,25). Therefore, in the present study, cTnI and CK-MB were utilized as indicators in order to observe the impact of dexmedetomidine on myocardial injuries. The results revealed that dexmedetomidine was able to reduce the plasma levels of cTnI and CK-MB in patients who underwent OPCAB surgery during dexmedetomidine administration. A reduction in the incidence of myocardial ischemia and arrhythmic events was observed, which indicated that dexmedetomidine was able to reduce post-surgical myocardial cell injury in patients undergoing OPCAB surgery. This result was consistent with the hypothesis for the study.

The techniques used in OPCAB surgery may lead to partial myocardial cell injury (26). Myocardial cell injury predominantly occurs due to the relative reduction in the myocardial oxygen supply during vascular anastomosis grafting, a lack of perfusion, leading to myocardial ischemia, or an ischemia reperfusion injury, all of which reduce the heart rate and myocardial contractile force during vascular anastomosis grafting. Vascular anastomosis grafting to reduce oxygen consumption is one of the main measures performed to reduce myocardial ischemia injury (4,27). In the present study, a dynamic electrocardiogram was obtained 72 h post-surgically, and 15 and six patients were observed to have myocardial ischemia in groups C and Dex, respectively. This result indicated that in the experimental conditions of this study, dexmedetomidine was able to reduce the incidence of post-surgical myocardial ischemic events, which may be a primary mechanism in reducing post-surgical myocardial cell injury.

The potential reduction in myocardial ischemia due to dexmedetomidine may be associated with a variety of factors. For example, the method of administration in this study was altered, compared with standard practice. The recommended method of administration is a load of 1 *μ*g/kg and an infusion time of >10 min, followed by an additional 0.2–0.7 *μ*g/kg/h for maintenance (28). However, high blood pressure and bradycardia occur at this load; thus, many clinicians suggest avoiding the recommended load, in order to reduce the adverse effects that dexmedetomidine may induce in the hemodynamics of patients with severe cardiovascular complications (29). The α_2_-receptor agonist, dexmedetomidine, may shrink the coronary arteries and reduce coronary blood flow (30,31). Blood flow is further reduced in patients with atherosclerosis, which may result in myocardial ischemia (32). Patients who undergo OPCAB surgery often have complications, such as hypertension, hyperlipidemia, atherosclerosis and diabetes mellitus. The synergistic effect of anesthesia-inducing drugs and dexmedetomidine may adversely influence ischemic myocardium. During the surgery, cardiac function has been demonstrated to considerably improve following the first vascular grafting (primarily for the anterior descending branches), as evidenced by the enhanced myocardial contractile force and a rise in blood pressure. Therefore, in this study, the patients were administered a continuous intravenous fusion of 0.2–0.5 *μ*g/kg/h dexmedetomidine following the first vascular grafting, in order to avoid the adverse effects of hemodynamic fluctuation on myocardial ischemia.

Dexmedetomidine may decrease the heart rate and blood pressure of patients who undergo surgery while on medication, thus reducing myocardial oxygen consumption. In addition to the mechanical cell injuries that occur as a result of the surgery itself (26), heart rate, blood pressure and vascular anastomosis time are the predominant factors that lead to myocardial cell injury during OPCAB. Low or no perfusion during vascular anastomosis results in certain regions of the myocardia exhibiting a hypoxic-ischemic status, which leads to ischemia or ischemia-reperfusion injury. When this occurs, reducing the heart rate and myocardial contractile force using β-receptor blockers may reduce myocardial oxygen consumption, which is critical in reducing ischemic injury (4,27). Dexmedetomidine treatment results in a sympathetic blockade, as it affects the α_2_-receptors in the central nervous system, restrains the release of the central sympathetic neurotransmitter (predominantly norepinephrine), reduces the peripheral sympathetic nervous tension and simultaneously excites the vagus nerve to reduce the heart rate (33). In the present study, the heart rate and blood pressure of the patients in group Dex were significantly lower than those in group C when the dexmedetomidine was administered. When the heart rate decreases, the post-surgical cardiac oxygen demand also decreases, thus facilitating the maintenance of the myocardial oxygen supply and demand balance. Therefore, these effects not only protect the ischemic myocardium prior to grafting, but also reduce the post-surgical incidence of myocardial injury. In a study where dexmedetomidine was applied to volunteers, an excessive infusion dosage or a very rapid infusion rate was observed to lead to a biphasic blood pressure response or a transient increase in blood pressure (particularly during the load period). When the plasma level of dexmedetomidine decreased, the blood pressure dropped and remained at lower levels (34). No increase in blood pressure was observed in this study; by contrast, the blood pressure decreased and remained stable with the application of dexmedetomidine, without violent fluctuation. Thus, cardiac afterload, myocardial oxygen consumption and myocardial ischemia injury all decreased during the surgery.

In 1997, Jalonen *et al* (15) observed 40 patients who underwent CABG surgery under CPB. This study revealed that dexmedetomidine was not able to reduce the incidence of myocardial ischemic events, which is inconsistent with the results obtained in the current study. There are a number of possible explanations for this contradiction.

In the study by Jalonen *et al*, all patients underwent CPB surgery, which is different from the surgical approach used in the current study. Many researchers have indicated that the incidence of myocardial injury resulting from OPCAB surgery was far lower than that resulting from CPB surgery (35). In addition, the methods of administration were different. In this study, dexmedetomidine was administered prior to the first vascular grafting until 12 h following the surgery, without the load dosage being used. However, in the previous study, 50 ng/kg/min dexmedetomidine was administered prior to anesthesia, with the administration lasting for 30 min, followed by 7 ng/kg/min dexmedetomidine, which was maintained until the end of the surgery. Furthermore, the observation method and time of ischemic events were different. In the current study, the dynamic electrocardiogram was monitored from the end of the surgery, until 72 h post-surgery, whereas in the previous study the dynamic electrocardiogram was monitored from 8 h prior to surgery, until 24 h post-surgery, and the presence of myocardial ischemia was also monitored seven days later using the dynamic electrocardiogram. These differences may have lead to different observation results.

Following CABG surgery, various types of arrhythmia, which are risk factors for the incidence of post-surgical adverse cardiac events subsequent to OPCAB surgery, influence postoperative hemodynamic stability. The excitation of the sympathetic nerve is the primary pathogenesis of tachyarrhythmia following OPCAB surgery (36). In a study by Kalman *et al* (37) a significant increase was observed in the incidence rate of atrial fibrillation when norepinephrine was intravenously injected into the patients to simulate excitation of the sympathetic nerve. In the test conditions of the present study, the norepinephrine level in group Dex was significantly lower than that in group C, and the incidence of arrhythmia in group Dex was lower than that in group C, particularly with regard to tachyarrhythmia, atrial fibrillation and ventricular arrhythmia. This result indicated that dexmedetomidine was able to inhibit the activity of the sympathetic nerve (similar to a reduction in the plasma level of norepinephrine) and reduce the incidence of arrhythmia. The impact of dexmedetomidine on cortisol was simultaneously observed in this study. The plasma level of cortisol decreased during the application of dexmedetomidine, which indicated that dexmedetomidine was inhibited in the sympathetic nerve. A number of other studies have also observed that dexmedetomidine may be applied in the treatment of arrhythmia during cardiac surgery. Chrysostomou *et al* (38) observed 14 patients with congenital heart diseases who had undergone surgery. The types of arrhythmia included ectopic junctional tachycardia, ectopic atrial tachycardia, reentrant supraventricular tachycardia, atrial flutter and rapid junctional rhythm. As a therapeutic drug or as a remedial drug when other anti-arrhythmic drugs failed (in nine and five cases, respectively), dexmedetomidine was observed to control the heart rate effectively or to transform the abnormal heart rate to sinus rhythm. These results indicated that dexmedetomidine may be applied in the treatment of acute supraventricular tachycardia during the surgery of patients with congenital heart disease. Furthermore, Ruesch and Levy (19) revealed a case where sustained tachycardia during OPCAB surgery was successfully treated using dexmedetomidine. However, the effects of dexmedetomidine as an anti-arrhythmic drug have rarely been investigated in clinical studies. Thus, further studies are required to evaluate its safety and efficacy.

There were certain limitations in this study. The administration times of dexmedetomidine were limited to intraoperatively and 12 h subsequent to surgery. Thus, the impact of prolonged administration on myocardial injuries was not observed. In addition, the long-term survival rates of the patients who underwent the surgery were not included in this study due to the time limitations. However, this is something to be studied in the future.

In conclusion, the mechanism by which dexmedetomidine reduced postoperative myocardial injuries in patients who had undergone OPCAB surgery was associated with the reduction of post-surgical myocardial ischemia and the reduced incidence of arrhythmia.

## Figures and Tables

**Table I.** Demographic data of the two groups concerning age, gender and preoperative therapeutic medicine (n=81 per group).

Group	Age (years)	Gender (male %)	Hypertension (%)	Hypercholesterolemia (%)	ACE inhibitors (%)	β-receptor blockers (%)	Calcium antagonists (%)	Nitrates (%)
C	58±6	34	79	67	60	89	29	90
Dex	60±4	31	81	70	58	91	34	92

The data for age are presented as the mean ±standard deviation. There are no significant differences between the two groups with respect to the demographic data (P>0.05). Group C, control group; Group Dex, dexmedetomidine-treated group; ACE, angiotensin-converting-enzyme.

**Table II.** Variation of blood pressure and heart rate at each time point (n=81 per group).

Variable	Group	T0	T1	T2	T3	T4	T5
SBP (mmHg)	C	136±14	101±13^a^	113±11^a^	123±10	134±12	131±11
	Dex	135±11	89±9^a^^b^	105±9^a^^b^	119±12	133±17	128±21
DBP (mmHg)	C	77±12	69±7^a^	72±11^a^	73±11	66±8	63±11
	Dex	80±4	62±10^a^^b^	65±11^a^^b^	70±11^a^	65±4^a^	59±11^a^
HR (bpm)	C	69±10	90±14	86±12	90±11	83±15	79±21
	Dex	76±8	66±12^a^^b^	64±15^a^^b^	79±12	76±14	73±11

Data are presented as the mean ± standard deviation.

a P<0.05 compared with T0;

b P<0.05 compared with the control group (Group C). T0, immediately prior to surgery; T1, immediately following surgery; T2, 12 h post-surgery; T3, 24 h post-surgery; T4, 48 h post-surgery; T5, 72 h post-surgery; SBP, systolic blood pressure; DBP, diastolic blood pressure; HR, heart rate; Group Dex, dexmedetomidine-treated group.

**Table III.** Plasma concentrations of norepinephrine and cortisol (n=81 per group).

Substance	Group	T0	T1	T2	T3	T4	T5
NE (ng/ml)	C	223.19±56.25	352.55±45.30^a^	398.53±82.53^a^	392.01±34.51^a^	395.71±92.00^a^	285.41±47.01^a^
	Dex	230.34±57.07	101.94±86.52^a^^b^	122.65±56.81^a^^b^	389.09±65.38^a^^c^	383.52±78.26^a^^c^	279.54±59.40^a^^c^
Cor (ng/ml)	C	257.50±51.81	223.70±56.80^a^	220.70±36.66^a^	378.70±26.35^a^^c^	298.90±46.30^a^^c^	279.40±50.70^a^^c^
	Dex	261.78±50.89	104.70±16.84^a^^b^	121.70±46.20^a^^b^	369.30±46.20^c^	289.70±29.80^c^	276.70±36.20

Data are presented as the mean ± standard deviation.

a P<0.05 compared with T0;

b P<0.05 compared with the control group (Group C);

c P<0.05 compared with T0, T1 and T2. T0, immediately prior to surgery; T1, immediately following surgery; T2, 12 h post-surgery; T3, 24 h post-surgery; T4, 48 h post-surgery; T5, 72 h post-surgery; Group Dex, dexmedetomidine-treated group; NE, norepinephrine; Cor, cortisol.

**Table IV.** Plasma concentrations of cardiac troponin I (cTnI) and creatine kinase-MB (CK-MB) at each time point (n=81 per group).

Substance	Group	T0	T1	T2	T3	T4	T5
cTnI (ng/ml)	C	0.19±0.02	3.55±0.53^a^	2.93±0.53^a^	2.61±0.51^a^	1.71±0.42^a^	1.41±0.27^a^
	Dex	0.16±0.07	2.14±0.52^a^	2.25±0.81^a^	2.09±0.38^a^^b^	1.12±0.26^a^^b^	0.54±0.40^a^^b^
CK-MB (ng/ml)	C	3.05±1.04	29.26±8.01^a^	39.40±21.05^a^	31.01±19.01^a^	26.39±10.15^a^	14.44±2.88^a^
	Dex	2.16±0.56	23.45±5.92^a^^b^	28.86±14.00^a^^b^	26.94±13.39^a^^b^	25.42±9.19^a^	15.67±3.32^a^

Data are presented as the mean ± standard deviation.

a P<0.05 compared with T0;

b P<0.05 compared with the control group (Group C). T1, immediately following surgery; T2, 12 h post-surgery; T3, 24 h post-surgery; T4, 48 h post-surgery; T5, 72 h post-surgery; Group C, control group; Group Dex, dexmedetomidine-treated group.
